# Applicability of Hydrogen Peroxide in Brown Tide Control – Culture and Microcosm Studies

**DOI:** 10.1371/journal.pone.0047844

**Published:** 2012-10-17

**Authors:** Varunpreet Randhawa, Megha Thakkar, Liping Wei

**Affiliations:** Department of Chemistry and Environmental Science, New Jersey Institute of Technology, Newark, New Jersey, United States of America; Dowling College, United States of America

## Abstract

Brown tide algal blooms, caused by the excessive growth of *Aureococcus anophagefferens*, recur in several northeastern US coastal bays. Direct bloom control could alleviate the ecological and economic damage associated with bloom outbreak. This paper explored the effectiveness and safety of natural chemical biocide hydrogen peroxide (H_2_O_2_) for brown tide bloom control. Culture studies showed that H_2_O_2_ at 1.6 mg L^−1^ effectively eradicated high density *A. anophagefferens* within 24-hr, but caused no significant growth inhibition in the diatoms, prymnesiophytes, green algae and dinoflagellates of >2–3 μm cell sizes among 12 phytoplankton species tested over 1-week observation. When applied to brown tide bloom prone natural seawater in a microcosm study, this treatment effectively removed the developing brown tide bloom, while the rest of phytoplankton assemblage (quantified via HPLC based marker pigment analyses), particularly the diatoms and green algae, experienced only transient suppression then recovered with total chlorophyll a exceeding that in the controls within 72-hr; cyanobacteria was not eradicated but was still reduced about 50% at 72-hr, as compared to the controls. The action of H_2_O_2_ against phytoplankton as a function of cell size and cell wall structure, and a realistic scenario of H_2_O_2_ application were discussed.

## Introduction

Harmful algal bloom (HAB) is a growing global problem, and in recent decades it has been seen increasing not only in the bloom incidences, but also in harmful species, affected areas, impacted fisheries resources and economic losses [1], [2], [3]. Direct bloom control refers to actions taken to suppress or destroy HABs – to directly intervene the bloom process [2], [4]. Among the physical, chemical and biological methods, only clay flocculation/sedimentation is well explored with reported field application successes in some Asian countries [5], [6]. Chemical methods have not been actively explored for algal bloom control, partly because of concerns on environmentally acceptability of the chemicals, and the potential side effects on other organisms [1], [2], [4]. The goal of this study is to explore the capability of one of the natural chemical biocide, hydrogen peroxide (H_2_O_2_), to effectively yet safely control brown tide algal blooms.

The brown tide algal blooms in northeastern US coastal bays are caused by the excessive growth of Pelagophyte *Aureococcus anophagefferens*, a species of unknown existence prior to the massive outbreak in 1985 [7]. The organism is named after its golden brown color, minute spherical cell shape (about 2 μm diameter), and toxin release that causes feeding cessation of a number of filter feeders [8]. During the bloom high cell density (e.g. 10^6^ cell mL^−1^) of *A. anophagefferens* discolors the water, causes severe light attenuation and subsequent decline of the submerged aquatic vegetations (SAV). The toxin(s) secretion, thought to be associated with *A. anophagefferens* exopolymer on cell surface, affects shellfish and zooplankton feeding behavior [7], [9]. The decline of SAV, which serves as the habitat and nursery ground for juvenile shellfishes, together with the feeding cessation response in juvenile shellfish species had devastated the commercial shellfishery in the affected area, such as the hard clams in New Jersey, bay scallop in New York, blue mussels in Rhode Island [7], [9]. Fortunately, in some of the brown tides recurring bays, the brown tides were localized with the possible initiation sites identifiable, for example, the Peconic Sound and Shennecock Bay for East Long Island brown tides [1], and Manahawkin Bay for Barnegat Bay brown tides [10]. This makes direct brown tide bloom control possible [4].

Hydrogen peroxide (H_2_O_2_) is naturally produced in sunlit surface seawater from photochemical reaction of dissolved organic matter, oxygen and trace metals [11], [12]. Reported quantum yield ranged from 4200 to 2.1 μmol H_2_O_2_ mol^−1^ photons at <340 nm light [13], and in mid-summer day H_2_O_2_ was reported to potentially accumulate at 30–59 nM hr^−1^ for several hours in 0.2 μm filtered seawater in Narragansett Bay, Rhode Island [14]. In coastal marine recreational water of Southern California summer dry season H_2_O_2_ concentrations averaged 122±38 nM [15]. Steigenberger [16] reported surface H_2_O_2_ ranged from 21 to 123 nM along a meridional transect in the eastern Atlantic Ocean. Rain water also contains hydrogen peroxide; up to 57 µM, approximately 2 mg L^−1^, were observed in wet deposition in Bermuda Atlantic Time Series Stations [17]. On the other hand, hydrogen peroxide is rapidly decomposed into water and oxygen, via enzyme reactions mediated by microorganisms, trace metal catalysis, halide, organic carbon, and direct photochemical loss [17], [18], [19], [20].

Hydrogen peroxide has been commonly used in industry, agriculture, aquaculture and environmental and personal hygiene applications for its oxidizing and microbiocidal properties. In environmental field it has been used in drinking water and wastewater treatment [21], [22], [23], (including cyanobacterial toxin removal [24], [25], [26]) in situ chemical remediation and to enhance in situ bioremediation of contaminated water, soil and sediment [27]. In aquatic environment, it has been approved for freshwater aquaculture usage as an antifungal and antibacterial therapeutic agent for the prevention and control of mortalities associated with external fungal infections (saprolegniasis) in cultured fish and fish eggs [28], [29]. H_2_O_2_-based products, such as sodium percarbonate, has been used as aquatic herbicides and microbiocide to manage algae, cyanobacteria, fungi and microorganisms in water (e.g. Massachusetts [30] and New York State [31]). Cyanobacteria were found more sensitive to H_2_O_2_ than eukaryotic phytoplankton [18], [32]. Low dose hydrogen peroxide has been shown successful in controlling a cyanobacteria bloom and microcystin toxin caused by *Planktothrix agardhii* [33]. In the study of Matthhijs et al. a special dispersal device (water harrow) was used to disperse H_2_O_2_ to the lake at 2 mg L^−1^, it resulted in more than 99% reduction of bloom organism of *Planktothrix agardhii* and algal toxin microcystin within a few days, while the eukaryotic phytoplankton, zooplankton, and macrofauna remained largely unaffected [33].

This study was carried out to examine the effectiveness of hydrogen peroxide in brown tide bloom control, and the safety of its application for the other and possibly co-occurring phytoplankton species. We first established a dose that effectively removed laboratory cultures of brown tide alga *A. anophagefferens*; then compared the H_2_O_2_ susceptibility of *A. anophagefferens* with eleven other marine phytoplankton species. Next, the response of the natural phytoplankton community to H_2_O_2_ exposure was examined by analyzing photosynthetic pigments of the algal community using high performance liquid chromatography (HPLC). The results provided an initial assessment on the effectiveness and safety of hydrogen peroxide in brown tide bloom control.

## Materials and Methods

### Algae species and culture conditions

Phytoplankton species investigated in this study were obtained from Provasoli-Guillard National Centre for Marine Phytoplankton (CCMP; now NCMA) and included the brown tide bloom alga *Aureococcus anophagefferens (Aa)*, five diatoms *Phaeodactylum tricornutum (Pt), Minutocellus polymorphus (Mpo)*, *Thalassiosira pseudonana (Tp)*, *Skeletonema costatum (Sc)*, and *Thalassiosira weissflogii (Tw)*, two green algae *Micromonas pusilla (Mp)* and *Dunaliella tertiolecta (Dt)*, two dinoflagellates *Amphidinium carterae (Ac)* and *Prorocentrum micans (Pm)*, and two Prymnesiophytes *Isochrysis galbana* and *Emiliania huxleyi* (Table 1). These species are common in marine waters, among them green algae *Mp* were found in abundance in non-bloom and postbloom waters [8], [34], and the diatoms *Pt* and *Tp* were found to co-exist with brown tide bloom in Quantuck Bay, NY [35], *Mpo*, *Tp*, *Sc* and *Pm* were also found in pre-bloom or post-bloom waters [9], [34]. These species were maintained in artificial seawater Aquil or Aquil-Si (Aquil without silicate addition, for species other than diatoms), which were prepared and sterilized according to Price et al [36]. Polycarbonate bottles (Nalgene, cleaned with detergent and acid) were used as culture vessels. All cultures were incubated in a diurnal growth chamber with 120 µE m^−2^ s^−1^ illumination from cool white fluorescence bulbs at 12∶12-h light-dark cycle, and 19±1°C.

**Table 1:** Marine phytoplankton species used in the study, listed group-wise and in order of increasing size.

Species	Class^e^	Cell size^e^ (µm)	CCMP Strain no.	Features
*Aureococcus anophagefferens*	Pelagophyceae	2–4	1984	Small spherical non-motile cell, no cell wall but a diffuse polysaccharide layer [8]
*Micromonas pusilla* a	Prasinophyceae	2–3 x 2–4	1545	Small green flagellate with organic scales [81], naked cell [82]
*Dunaliella tertiolecta* a	Chlorophyceae	6–9	1320	Green flagellate, no cell wall but mucilage layer [81]
*Phaeodactylum tricornutum* ^b^	Bacillariophyceae	2–4 x 12–14	1327	Pennate diatom, weakly siliceous cell wall [81]
*Minutocellus polymorphus* ^b^	Coscinodiscophyceae	3–6	499	Centric diatom, siliceous cell wall [81]
*Thalassiosira pseudonana* ^b^	Coscinodiscophyceae	4–5 x 4–6	1335	Centric diatom, siliceous cell wall
*Skeletonema costatum* ^b^	Coscinodiscophyceae	4–5 x 4–6	2092	Centric diatom, Siliceous cell wall [47]
*Thalassiosira weissflogii* ^b^	Coscinodiscophyceae	10–12 x 12–22	1335	Centric diatom, siliceous cell wall [47]
*Isochrysis galbana* ^c^	Prymnesiophyceae	2–4 x 4–6	1323	Flagellate, no cell wall [49], [50]
*Emiliania huxleyi* ^c^	Prymnesiophyceae	4–6 x 4–8	374	Coccolith absent^ e^
*Amphidinium carterae* ^d^	Dinophyceae	9–13 x 12–18	1314	Dinoflagellate, thecal plate absent [83]
*Prorocentrum micans* ^d^	Dinophyceae	25–30 x 35–45	1589	Dinoflagellate, cellulose thecal plate present [83]

a Green algae, ^b^Diatoms, ^c^Prymnesiophytes, ^d^Dinoflagellates. **^e^** https://ncma.bigelow.org/strain information.

### H_2_O_2_ exposure and the quantification of algal growth response

Hydrogen peroxide (Fluka-95321, *Trace*SELECT®, 30% w:w, 9.8 M, kept in refrigerator and in dark) was first diluted with sterile culture medium to 10 mM (∼0.03%), then immediately added to the mid- or late-exponential phase cultures. Upon gentle mixing, the cultures (50 mL in 250 mL Nalgene polycarbonate bottles) were returned to the growth chamber for incubation. The nominal H_2_O_2_ addition was 0, 0.8, 1.6, 3.2 and 6.4 mg L^−1^ for *A. anophagefferens*, and 0, 1.6 and 6.4 mg L^−1^ for other algal species. The nominal concentrations of hydrogen peroxide were confirmed with 10–15% accuracy using spectrophotometric method of Lu et al. [37], where H_2_O_2_ reacted with p-nitrophenylboronic acid stoichiometrically at pH 9 producing p-nitrophenol of 19400 cm^−1^ M^−1^ absorptivity. Cultures without H_2_O_2_ addition were used as controls. Cultures were in triplicates for *A. anophagefferens*, and duplicates for other species.

Parameters reflecting algal growth were monitored, typically at 0 hr (immediately before adding H_2_O_2_), 3 hr, and then daily up to 1 week after H_2_O_2_ addition. The *in vivo* fluorescence (IVF) was monitored using Turner Designs' Trilogy Fluorometer (equipped with an optical block for *in vivo* chlorophyll a measurement, excitation 485 nm, emission 685 nm, band width 50 nm). Cell density was measured by Coulter Counter Multisizer 3 (equipped with a 70 μm aperture tube, Beckman). Total chlorophyll a (chl a) was measured with GF/F filtration, 90% acetone extraction, and UV-Vis spectrophotometer (Agilent 8453) quantification using Jeffery and Humphrey's trichromatic equation following EPA method 446.0.

### Statistical analysis

Throughout the study Minitab 16 Software Package was used in statistical analysis with probability *p*<0.05 being accepted as statistically significant. Two-sample t-tests were used to compare the control cultures and the H_2_O_2_ treated cultures. Paired t-tests and/or ANOVA (analysis of variance) were used to compare temporal variation of the cultures. Exponential growth or log-linearity was determined with regression analysis. Regression was also used to determine the correlation between percent inhibition and both cell size and H_2_O_2_ dose.

### Microcosm study

Natural seawater was collected from Barnegat Bay, NJ, which is prone to summer brown tide, by pumping surface seawater into 20-L carboy (pre-cleaned with detergent and acid) from a boat on July 28, 2010. (No specific permission is required to collect seawater from Barnegat Bay, NJ, as it is not privately-owned nor protected in any way. The work does not involve endangered or protected species.) Collected seawater was kept in a cooler and stored in cold-room (4°C) for processing next day. The upper Barnegat Bay summer phytoplankton assemblage typically consists of small coccoidal picophytoplankton complex (<5 μm) *Aureococcus-Chlorella-Nannochloris-Synechococcus* complex, other small flagellates of 3–7 μm size (chrysophyte e.g. *Calycomonas oval*, prasionophyte e.g. *Micromonas sp.*), prymnesiophytes such as *Chrysochromulia sp.*, diatoms of small or large sizes (e.g. *Minutocella polymorphus, Thalasiosira sp., Cyclotella sp)*, and small numbers of larger species (>15 μm) e.g. eugalenophyte *Eugalena sp*, and raphidophyte *Heterosigma carterae* [34].

In order to test cell size dependency, if any, of H_2_O_2_ action, a fraction of the collected natural seawater was passed through 5 µm PTFE membrane filters (Millipore) under low vacuum pressure (<5 psi). Unfiltered and 5 µm filtered seawater microcosms were set up by distributing 450 mL seawater into 500 mL sterilized polycarbonate bottles, and subsequently inoculating 35,000 cell mL^−1^ cultured *A. anophagefferens* (to mimic a medium density brown tide bloom) [10]. H_2_O_2_, freshly prepared in sterile Aquil at 10 mM, was added at 1.6 and 6.4 mg L^−1^. The controls (microcosms without H_2_O_2_ addition) and 1.6 mg L^−1^ H_2_O_2_ treatments were in triplicates; microcosms with 6.4 mg L^−1^ H_2_O_2_ addition were not replicated. The microcosms were incubated in the growth chamber as in the culture study.

Total phytoplankton growth, *A. anophagefferens* cell density, and phytoplankton community composition were analyzed at 0 hr (immediately before H_2_O_2_ addition), and then daily up to 72 hrs of H_2_O_2_ addition. Total phytoplankton growth was analyzed via *in vivo* chlorophyll fluorescence and total chl a (same as in culture study). Changes in phytoplankton community composition were tracked via accessory pigment analysis: 19′-butanoyloxyfucoxanthin (19′-bf) for brown tide alga *A. anophagefferens* [38], fucoxanthin for diatoms and other chrysophytes, chlorophyll b (chl b) for chlorophytes and prasinophytes, peridinin for dinoflagellates, and zeaxanthin for cyanobacteria, respectively [39], [40]. *A. anophagefferens* cell density was further estimated from a standard curve that correlated the number of cultured *A. anophagefferens* cells used in the pigment analysis and the resulting chromatographic peak area of 19′-bf.

Accessory pigment analysis followed Van Heukelem and Thomas' HPLC method [41]. Briefly, 75 mL microcosm water sample was filtered through GF/F (nominal pore size 0.7 μm, 25 mm, Whatman, vacuum pressure <5 psi), and the filters were immediately preserved in liquid nitrogen. During analysis, the filters were put in 2-mL 90% acetone in amber micro-centrifuge tubes (VWR 20170–084) and sonicated in ice-cold water bath for 30 minutes (Ultrasonic FS-28, Fischer Scientific) to extract the pigments. The acetone extracts were centrifuged and the supernatants filtered through 0.45 µm PTFE syringe filters (4 mm, Pall Life Sciences) for HPLC analysis. These operations were in dim lights to minimize photo-degradation of the pigments. A Waters HPLC system, equipped with Waters 2690 separation module, refrigerated auto sampler, Eclipse XDB C8 column (150 x 4.6 mm, 3.5 µm, Agilent) and guard column, column heater, Waters 486 UV-VIS absorbance detector set at 450 nm, and SRI Model 333 USB Chromatography Data System, was used in pigment analysis following Van Heukelem and Thomas [41]. Pigments were identified by comparing the retention times of the standards, including chlorophylls a, b, β-carotene (Sigma Aldrich), and fucoxanthin (DHI Group, Denmark). Other pigments were identified by comparison with the culture extracts from *A. anophagefferens*, *Amphidinium carterae, Synechococcus sp*. and *Dunaliella tertiolecta* for 19′-bf, peridinin, zeaxanthin and chl b respectively.

## Results

### Effect of hydrogen peroxide on *A. anophagefferens* growth

**Figure 1:**
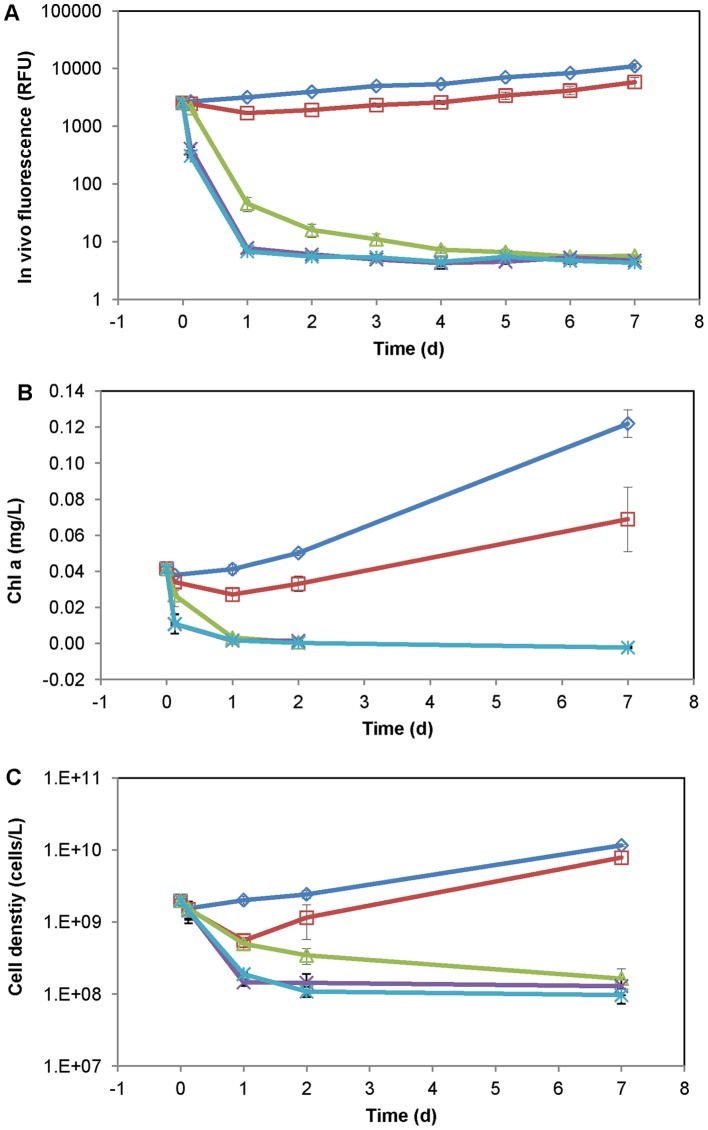
Culture study *Aureococcus anophagefferens* growth response. Response of *Aureococcus anophagefferens* cultures to the H_2_O_2_ addition at 0 (control, ◊), 0.8 (□), 1.6 (Δ), 3.2 (x) and 6.4 (*) mg L^−1^. A) *In vivo* chlorophyll a fluorescence. B) Total chl a. C) Cell density. Vertical error bars represent standard deviations of triplicate cultures.

Hydrogen peroxide at 1.6 mg L^−1^ effectively inhibited *A. anophagefferens* growth as was observed with *in vivo* chlorophyll a fluorescence, total chl a, and cell density (Fig. 1). The inhibition was transient at 0.8 mg L^−1^ but was lasting at ≥1.6 mg L^−1^ H_2_O_2_ (Fig. 1). The starting cultures were in the late exponential growth phase, with *in vivo* fluorescence averaged 2565±35 (relative fluorescence unit, 15 cultures), total chl a 41.5±1.2 μg L^−1^, and cell density 1.98±0.4 million cells mL^−1^. The control cultures experienced 4.5-fold log-linear increase in *in vivo* fluorescence during 1-week incubation (Ln F  = 7.87+0.20 t, where F is the *in vivo* fluorescence, t is time in days, R^2^  = 0.989, *p* = 0.000), but the cultures with 0.8 mg L^−1^ H_2_O_2_ addition had a decrease in *in vivo* fluorescence nearly 50% at 24-hr (*p* = 0.001) which then rebounded at a rate comparable to that of the control cultures (*p* = 0.975). More importantly, the cultures with 1.6, 3.2 and 6.4 mg L^−1^ H_2_O_2_ addition showed 98%, 99.7% and 99.7% (*p* = 0.000) decrease in *in vivo* fluorescence at 24-hr and re-growth was not observed over 1 week (Fig. 1A). Total chl a in these cultures (with 1.6, 3.2 and 6.4 mg L^−1^ H_2_O_2_) decreased more than 90% (*p*≤0.002) at 24-hr, and were below detection limit at 1-week (Fig. 1B). In the cultures with 1.6 mg L^−1^ H_2_O_2_ addition, cell density decreased log-linearly (LOG cell/L  = 9.06–0.138 t, where t is time in days, *p* = 0.000) from nearly 2 to 0.5, 0.34 and 0.16 million cells mL^−1^at 24-hr, 48-hr and 1-week respectively; and in the cultures with 3.2 and 6.4 mg L^−1^ H_2_O_2_ addition, it decreased >90% to below 0.19±0.02 million cells mL^−1^at and after 24-hr (Fig. 1C).

Medium effective concentration, EC_50_, was calculated from *in vivo* fluorescence based on the logistic model 
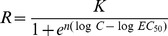
 where R is growth response (% inhibition on *in vivo* fluorescence relative to the controls), K is the growth response of the control (K = 1), C is H_2_O_2_ concentration (mg L^−1^), EC_50_ is medium effective concentration. The resulting EC_50_s for *A. anophagefferens* were 1.39±0.06 mg L^−1^ at 3-hr, and 0.91±0.00 mg L^−1^ at 24-hr and remained at this level during 48-hr –1-week.

Overall, *A. anophagefferens* growth response suggested that H_2_O_2_ at 1.6 mg L^−1^ or higher concentrations effectively removed high density *A. anophagefferens* within 24-hr exposure (>90% in total chl a), and re-growth was not observable during 1-week incubation.

### Growth response of other phytoplankton species to H_2_O_2_ exposure

Because effective (>90% in total chl a) *A. anophagefferens* removal was accomplished by 1.6 mg L^−1^ H_2_O_2_ treatment, comparisons on the survival and re-growth of *A. anophagefferens* with 11 other marine phytoplankton species (5 diatoms *Pt, Mpo, Tp, Sc, Tw*; 2 green algae *Mp* and *Dt*, 2 prymnesiophytes I*g* and *Eh*, and 2 dinoflagellates *Ac* and *Pm*) were carried out, by exposing the growing or grown cultures of similar *in vivo* chlorophyll fluorescence (thus similar initial biomass) to 1.6 mg L^−1^ H_2_O_2_.

**Figure 2:**
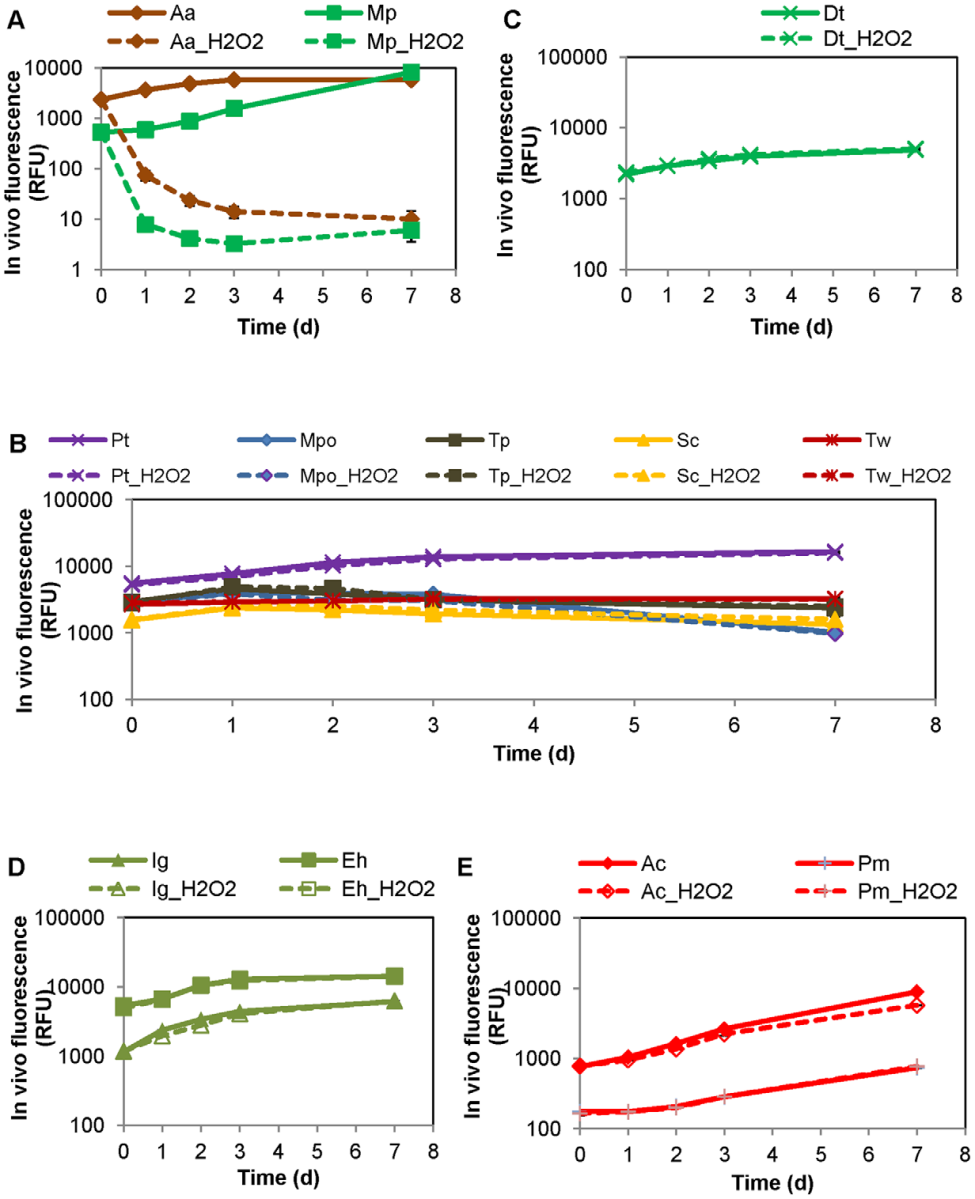
Response of various phytoplankton species in culture study. Variation of *in vivo fluorescence* of 12 marine phytoplankton species upon H_2_O_2_ addition at 0 (control, solid line) and 1.6 mg L^−1^ (treatment, dashed line). A) *Aureococcus anophagefferens* (*Aa*) and prasinophyte *Micromonas pusilla* (*Mp*). B) Diatoms *Phaeodactylum tricornutum* (*Pt*), *Minutocellus polymorphus* (*Mpo*), *Skeletonema costatum* (*Sc*), *Thalassiosira pseudonana* (*Tp*) and *Thalassiosira weissflogii* (*Tw*). C) Chlorophyte *Dunaliella tertiolecta* (*Dt*). D) Prymnesiophytes *Isochrysis galbana* (*Ig*) and *Emiliania huxleyi* (*Eh*). E) Dinoflagellates *Amphidinium carterae* (*Ac*) and *Prorocentrum micans* (*Pm*). Vertical error bars are deviations between duplicate cultures.

Among these 11 phytoplankton species only *Micromonas pusilla (Mp)* (a very small green algae, Class of Prasinophyceae, Phylum of Chlorophyta) was eradicated by 1.6 mg L^−1^ H_2_O_2_ (Fig. 2). The *in vivo* fluorescence of *Mp* decreased >90% within 24-hr of 1.6 mg L^−1^ H_2_O_2_ addition, and remained low during the 1-week observation, similar to *A. anophagefferens* tested in parallel (Fig. 2A). A toxic dinoflagellate, *A. carterae (Ac)*, had an overall 17% lower growth rate as compared to its control cultures (*p* = 0.018, Fig. 2E).

The growth of the rest 9 species, i.e. the diatoms *Pt, Mpo, Tp, Sc, Tw* (Fig. 2B), the other green algae *Dt* (Fig. 2C), the prymnesiophytes *Ig* & *Eh* (Fig. 2D), and the dinoflagellate *Pm* (Fig. 2E), were not affected by 1.6 mg L^−1^ H_2_O_2_ addition, as compared to the respective controls (*p*>0.05, two-sample t-tests on growth rates for *Pt, Ig, Dt* and *Pm*, which grew exponentially both with and without H_2_O_2_ addition, and two-sample t-tests on *in vivo* fluorescence at each time point for *Mpo, Tp, Sc, Tw, Eh* which did not grow exponentially during the 1-week observation. The exponential growth status was determined with log-linearity regression analysis with r ≥0.88, n = 4, *p*<0.05.).

Exposure of these species to H_2_O_2_ at 6.4 mg L^−1^ revealed cell size dependent species sensitivity (Table 2). It was found that the smaller the cell size the more sensitive it is to H_2_O_2_. For example, among the diatoms, with decreasing cell size (*Tw* >*Sc* >*Tp* >*Mpo* >*Pt*) the percent inhibition increased from non-inhibitive in *Sc* and *Tw* to more than 90% inhibition in *Pt* (Table 2). An empirical correlation was found statistically significant: % inhibition  = 0.688–0.0561 x cell diameter +0.00262 x H_2_O_2_/chl a (*p* = 0.005, n = 12, R^2^ = 0.69) where % inhibition is the average % inhibition at 24-hr, 48-hr and 72-hr upon 6.4 mg L^−1^ H_2_O_2_ addition as compared to the control, cell diameter is the mean cell size measured by Coulter counter (μm), and H_2_O_2_/chl a is the weight ratio, mg H_2_O_2_/mg chl a, at time zero. Since dinoflagellate *Pm* (45 μm) is substantially larger than the rest of the species (1.4–11.05 μm), a correlation of 11 species excluding *Pm* was also established, % inhibition  = 0.913–0.0952 x cell diameter +0.00224 x H_2_O_2_/chl a (*p* = 0.006, n = 11, R^2^ = 0.72), to reflect species sensitivity as a function of cell size and H_2_O_2_ dose. In this regression, the cell diameter alone was found to explain 40% of the variations.

**Table 2:** Mean cell diameter as measured by Coulter counter, and percent growth inhibition (mean ± standard deviation) at 24-hr, 48-hr and 72 hr after addition of 6.4 mg L^−1^ H_2_O_2_, as compared to the controls, of the 12 phytoplankton species tested.

		Green algae		Diatoms					Prymnesio-phytes		Dino-flagellates	
Species	*Aa*	*Mp*	*Dt*	*Pt*	*Mpo*	*Tp*	*Sc*	*Tw*	*Ig*	*Eh*	*Ac*	*Pm*
Cell diamter (μm)	1.83	1.4	5.5	3.4	3.5	3.8	6.6	11.05	3.6	3.7	9	45
% inhibition	99.8±0.2	99.4±0.4	1.3±8.1^a^	96.0±3.5	89.8±12.3	71.5±13.5	9.6±11.9 a	2.6±2.6^a^	98.6±0.9	95.9±4.9	95.7±6.4	11.2±8.5

a The inhibitive effect is not statistically significant.

### Phytoplankton community response to H_2_O_2_ exposure in microcosm study

**Figure 3:**
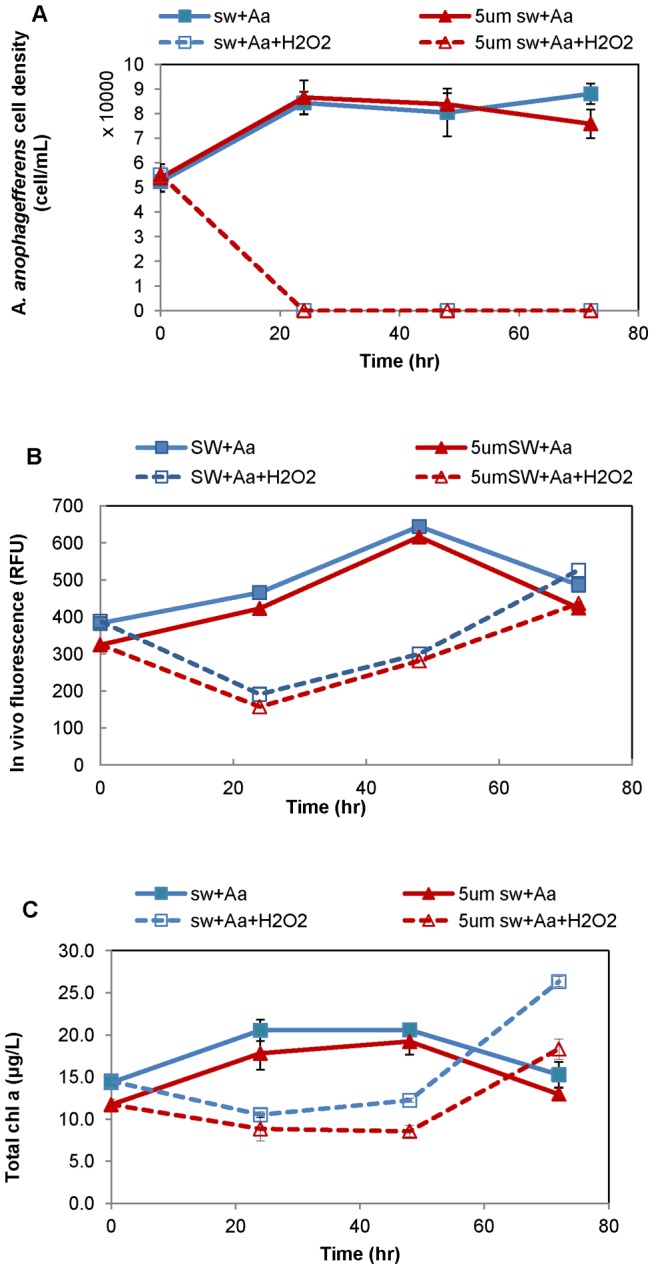
*Aureococcus anophagefferens* response in microcosm study. Variation in cell density of *Aureococcus anophagefferens* (A), *in vivo* chlorophyll fluorescence (B), and total chlorophyll a (C) in microcosms with 1.6 mg L^−1^ of H_2_O_2_ addition (treatment, dashed line) and without H_2_O_2_ addition (control, solid line). The microcosms were made of unfiltered (square symbols) and 5 μm filtered (triangle symbols) Barnegat Bay seawater amended with laboratory *A. anophagefferens* cultures. Vertical error bars represent standard deviations of triplicate microcosms.

*A. anophagefferens* cell density was estimated at 27,900±5,379 cell mL^−1^ (n = 2) in the original Barnegat Bay water samples, and at 54,150±3,188 cell mL^−1^ in the testing microcosms before H_2_O_2_ addition (n = 12, laboratory cultured *A. anophagefferens* was added to the microcosms). In the control microcosms (i.e. no H_2_O_2_ addition), *A. anophagefferens* cell density increased 60% at 24-hr as compared to time zero (*p*<0.05), and remained nearly constant at 24-hr, 48-hr and 72-hr (*p*>0.05, one way ANOVA, for both unfiltered and 5-μm filtered seawater microcosms), suggesting a developing brown tide bloom (Fig. 3A). In microcosms with 1.6 mg L^−1^ H_2_O_2_ addition, *A. anophagefferens* was eradicated (∼100%) at 24-hr, and its cell density remained low during the entire 72-hr study period, in both unfiltered and 5-μm filtered seawater microcosms (Fig. 3A).

While *A. anophagefferens* was eradicated in the phytoplankton assemblage, total phytoplankton productivity remained robust after a transient suppression at 24-hr and 48-hr (Fig. 3B). Specifically, after 1.6 mg L^−1^ H_2_O_2_ addition, the *in vivo* fluorescence was 50–60% lower than the controls at 24-hr and 48-hr (*p*≤0.001), but recovered, at 72-hr, to a level comparable to (*p* = 0.333, the 5 μm filtered microcosms,) or even higher (*p* = 0.035, the unfiltered microcosms) than those in the control microcosms (Fig. 3B). The transient initial suppression and subsequent recovery was also evident from total chl a. Total chl a was reduced 40–55% at 24-hr and 48-hr, as compared to the control, but recovered to exceed the control by 40% and 70% in 5 μm filtered and unfiltered microcosms, respectively (*p*<0.01) (Fig. 3C).

The response of the rest of the phytoplankton assemblage to 1.6 mg L^−1^ H_2_O_2_ treatment was estimated from algal marker pigments, fucoxanthin for diatoms and other chrysophytes, chl b for green algae, peridinin for dinoflagellates and zeaxanthin for cyanobacteria (Table 3). In the unfiltered seawater microcosms, fucoxanthin was reduced (by 1.6 mg L^−1^ H_2_O_2_) by 55% at 24-hr (*p* = 0.006) and 44% at 48-hr (*p* = 0.000), but subsequently recovered and exceeded the controls by 104% at 72-hr (*p* = 0.003); chl b was reduced approximately 55% at 24-hr and 48-hr (*p*<0.01) then recovered to a level not significantly different from the controls (*p* = 0.267) (Table 3). In the 5 μm filtered seawater microcosms, fucoxanthin was reduced about 58% at 24-and 48-hr (*p*<0.01) then exceeded the controls by 66% at 72-hr (*p* = 0.000); chl b was exceeded by the control by 54–67% at 24-hr and 48-hr (*p*<0.01) and then to a less degree of 17% at 72-hr (*p* = 0.018) (Table 3). Peridinin and zeaxanthin were not always detected; but where they were quantifiable in all triplicates, peridinin seemed to be reduced by 34% at 24-hr then recovered to a level comparable to the control at 48-hr in unfiltered seawater microcosms, and zeaxanthin was still reduced approximately 48% and 55% at 72-hr (*p*<0.05) in unfiltered and 5 μm filtered seawater microcosms, respectively (Table 3). Altogether above results suggest transient reduction but robust re-growth of diatoms and green algae in both pico, nano- and micro-phytoplankton communities.

**Table 3:** Percent decrease of the marker pigment concentrations (mean ± standard deviation of triplicate microcosms, and the probability) treated with 1.6 mg L^−1^ H_2_O_2_, as compared to the control without H_2_O_2_ addition, in microcosms with unfiltered and 5-μm filtered seawater.

	Fucoxanthin	Chl b	Peridinin	Zeaxanthin
**Microcosms with unfiltered seawater**				
0 hr	2±3% (0.248^a^)	3±4% (0.452)	N.A.	15±5% (0.023)
24 hr	55±2% (0.006)	53±2% (0.007)	34±5% (0.010)	17±12% (0.136)
48 hr	44±1% (0.000)	58±1% (0.001)	18±18% (0.292)	N.A.
72 hr	−104 ^b^±3% (0.003)	8±9% (0.018)	N.A.	48±8% 0.012)
**Microcosms with 5 μm filtered seawater**				
0 hr	4±4% (0.224)	0±6% (0.939)	22±15% (0.289)	0±9% (0.978)
24 hr	60±7% (0.002)	54±6% (0.003)	N.A.	N.A.
48 hr	57±5% (0.002)	67±2% (0.003)	N.A.	N.A.
72 hr	−66 ^b^±5% (0.000)	17±4% (0.018)	N.A.	55±4% (0.010)

a The values in parenthesis are probabilities of the two sample t-tests; *p*<0.05 suggests the percent change is statistically significant. ^b^ A negative percentage value indicates pigment concentrations were greater in treatment microcosms than the controls. N.A^.^ indicates pigment quantification might not be accurate as at least one of the triplicates went below detection limit.

Cell size dependent action of H_2_O_2_ was also evident, as the phytoplankton groups in 5 μm filtered seawater microcosms (dominated by picophytoplankton) experienced slightly greater percent inhibition and/or less robust recovery as compared to that in unfiltered seawater microcosms (which was a mix of pico-, nano- and microphytoplankton) (Table 3, and Fig. 3B and C).

A limited numbers of microcosms were exposed to 6.4 mg L^−1^ H_2_O_2_. As expected, 6.4 mg L^−1^ H_2_O_2_ completely removed 19′-bf pigment during the 72-hr study period, suggesting *A. anophagefferens* eradication. Chl a, fucoxanthin, chl b, peridinin were still exceeded by the controls at 72-hr by 64–71%, 93–97%, 5–28% and 49% respectively, and zeaxanthin went below detection limit during 24–72 hr (data not shown).

## Discussion

This study presented strong evidence, from culture and microcosm studies, that low dose of H_2_O_2_ (e.g. 1.6 mg L^−1^) effectively removed more than 90% of brown tide alga *Aureococcus anophagefferens* even at peak bloom density, without devastating most of the other potentially coexisting phytoplankton species and the natural phytoplankton community. Mechanisms of the differential susceptibility among phytoplankton and a realistic application scenario are discussed below.

### Differential phytoplankton susceptibility to hydrogen peroxide

*A. anophagefferens* demonstrated sensitivity similar to those of cyanobacteria. For example the EC_50_ were 0.3–0.4 mg L^−1^ for *Microcystis aeruginosa* and *Trichormus variabilis* and were between 1.1 and 1.7 mg L^−1^ for *Synechococcus nidulans* and two other cyanobacteria species on photosynthetic yield over 3-hr H_2_O_2_ exposure [42], in comparison with the EC_50_ of 1.39 mg L^−1^ on *in vivo* fluorescence based growth inhibition of *A. anophagefferens* over 3-hr exposure. Cyanobacteria has been shown to be more sensitive than most eukaryotic phytoplankton [18], [21], [32], [42], partly because of their prokaryotic photosynthetic apparatus [42]. The high sensitivity of *A. anophagefferens* to H_2_O_2_ may be attributed to both its small cell size and the fact that it lacks cell wall. *A. anophagefferens* has only a diffuse layer of polysaccharide external to plasma membrane [8]. As a result H_2_O_2_ can easily penetrate into the cell, and damage the cell by producing hydroxyl radicals which inhibit the photosynthetic electron transfer and the photosynthetic activity [42] among other effects.

The cell size dependent H_2_O_2_ sensitivity is likely caused by both the surface area to volume ratio being a function of cell size, and the non-selective reactivity of H_2_O_2_ and hydroxyl radical (·OH) product. The ·OH, a product of H_2_O_2_ interacting with ferrous ion via Fenton reactions [43] (Fe^2+^ may be formed via extracellular Fe(III) reduction [44]), is much more reactive than H_2_O_2._ It reacts with a wide range of organic compounds (alcohols, esters, unsaturated organics, aromatics, chlorinated hydrocarbons etc.) very rapidly with a second order reaction rate constant in the range of 10^7^–10^9^ M^−1^S^−1^ [27], [45]. The ·OH potentially initiates radical chain reactions such as hydrogen abstraction and double bond addition, causing damage to biomolecules and cellular components on cell membrane, chloroplast [18], [42] and DNA [46], eventually leading to oxidative stress and cell death. In terms of total cell surface area, cultures of the same biomass concentration but smaller cell size, as compared to larger-cell cultures, would have greater total surface area exposed to H_2_O_2_ and ·OH. As a result, the damage would be more extensive.

Other than cell size, the cellular structure, particularly the presence of rigid cell wall, may contribute to a species' H_2_O_2_ resistance. For example, the silica-mucilage cell wall of the diatoms [47], [48] may have given *Sc* and *Tw* their H_2_O_2_ resistance (diameter 6.6 and 11.05 μm respectively, not affected by 6.4 mg L^−1^ H_2_O_2_), as compared to the athecate dinoflagellate *Ac* of similar cell size (diameter 9 μm, but showed >90% inhibition by 6.4 mg L^−1^ H_2_O_2_) (Table 1 and 2). The prymnesiophytes *Ig* lacks cell wall (and is often used in aquaculture for easy assimilation by larval animals because of *Ig*'s small cell size and lack of tough cell wall) [49], [50], while *Eh* (CCMP 374) is non-coccolith forming strain (https://ncma.bigelow.org/, strain information); and both are more sensitive to H_2_O_2_ (3.6 and 3.7 μm respectively, showed >95% inhibition by 6.4 mg L^−1^ H_2_O_2_) than the diatoms of similar cell size e.g. *Mpo* (3.5 μm, 90% inhibition) and *Tp* (3.8 μm, 72% inhibition) (Table 1 and 2). The rigid cell wall, if present, potentially provides a first layer of defense against H_2_O_2_ and HO·, and allowing them to be inactivated before reaching the more sensitive and biologically critical components of cell membrane and organelles.

Likewise, Jeong et al. [51] found log-log relationship between the mortality rate of aquatic organisms (red tide dinoflagellates, diatoms, ciliates, copepods, fin-fish, brine shrimp and shellfish) and organisms' bio-volume, when exposed to hypochlorite, another small molecule oxidizing biocide. They further reported that diatoms were more resistant to hypochlorite than dinoflagellates even if they were smaller than the dinoflagellates in their study. This was attributed to siliceous cell wall and the large vacuole volume of diatom cells [51].

Hydrogen peroxide may be inactivated and detoxified by organisms' reactive oxygen species scavenging systems. Once H_2_O_2_ penetrates into a cell, reactive oxygen species scavenging systems would decompose and inactivate the excessive amounts of H_2_O_2_ [52], [53]. Glutathione peroxidase, ascorbate peroxidase [54], catalase [55], and various antioxidants such as ascorbate [53], glutathione [56] and even mycosporine and mycosporine-like amino acids [57], [58] likely participate in H_2_O_2_ and HO· scavenging. This, along with the capability of the cell to repair and replace the damaged biomolecules and cellular components, permits the culture to re-grow, as was observed in both the culture and the microcosm studies at relatively low H_2_O_2_ exposure.

### A consideration of a realistic scenario

Ex situ treatment of coastal algal bloom could be very costly given the enormous large size of water to be treated. For example if a bay water of 5×5 km^2^ with a depth of 1 m (2.5×10^10^ L or 6500 million gallons of water) is to be treated ex situ within 5 days, it would require a treatment capacity of 1300 million gallon per day (mgd), nearly 4 times that of Passaic Valley Sewage Commissioner, New Jersey, which is among the largest wastewater treatment facilities in Eastern US. Further, if the water is to be retained for 1 day before disposal, it would need 1300 million gallon reactor, representing an enormous size.

In situ treatment should be more feasible, although challenges exist. To treat brown tide bloom at 1.6 mg L^−1^ H_2_O_2_, one challenge is to disperse the concentrated H_2_O_2_ stock solution homogenously into seawater with minimal localized high concentration. In our study the stock solution (30% H_2_O_2_) was diluted 1000-fold before adding to the cultures. This dilution scheme requires an addition of 4.8-mm water containing 0.03% H_2_O_2_ to bloom water of 1-m depth (or 4.8 µm of 30% H_2_O_2_); and the wave action or a mixing device makes the additional 200-fold dilution to arrive at 1.6 mg L^−1^. The rate of chemical dispersal facilitated by wave action should be examined ahead of time, if possible, using hydrologic tracer chemicals such as sulfur hexafluoride (SF_6_) [59]. In Lake Koetshuis cyanobacterial bloom control Matthij et al. achieved nearly homogenous dispersal at 2 mg L^−1^ H_2_O_2_ using 10% H_2_O_2_ stock solution [33]. The mixing scheme was that computer controlled pumps (operated from a small boat) mixed 10% hydrogen peroxide stock solution with lake water at 500-fold dilution, which was then directly dispersed into the lake using a water harrow to obtain additional 100-fold dilution at 2 mg L^−1^ [33]. The homogeneity was verified within 3-hrs of dispersal [33]. Thus, this challenge is potentially solvable.

A second concern in in situ treatment is whether the efficacy of the treatment observed in the culture and microcosm studies is transferable in field applications, and further, what environmental factors may lead to variable success of bloom control. Since the OH radical is believed to be the active chemical species that attacks and kills the cells during H2O2 treatment, environmental factors that affect H2O2 decay and OH radical production likely also influence the efficacy of the treatment. Among other factors, Fe2^+^/Fe3^+^ and UV radiation could be important, as they would promote the conversion of H2O2 into OH radical through Fenton reaction (Fe2^+^ + H2O2 → Fe3^+^ + OH·+ OH−, Fe3^+^ + H2O2 → Fe2^+^ + OOH·+ H^+^) [60] and photolysis reaction (H2O2 + hγ  =  2 OH·) [61], respectively. In our culture study Fe3^+^ was well controlled at 6.6 × 10−19 M by using 100 µM EDTA and 8.3 µM total iron, and no Fe2^+^ was externally added [36]. In comparison, in natural seawater Fe3^+^ and total dissolved iron (FeT) vary over a range of 1–2 orders of magnitude. For example, Peconic Estuary (an urban estuary in Long Island Sound, NY) had FeT at 10–100 nM [62] and 28–237 nM averaging 114±81 nM, and Fe3^+^ at 1.1×10−20–4.7×10−19 M averaging 1.3x10−19 M, where salinity ≥26 parts per thousand [63]. Wu et al. reported 0.45–6.2 nM FeT in northwest Atlantic coastal water from the continental slope to near the mouth of Delaware Bay (6.2 nM) [64]. Oceanic surface seawater has lower FeT, typically at 0.02–1 nM [64]. FeT and Fe2^+^/Fe3^+^ were not measured in our microcosm sample taken from Barnegat Bay, NJ; but they could be similar to those of Peconic Estuary, based on reports of FeT in river water that discharges to these estuaries, e.g. 926 µg L−1 in Metedeconk River in Aug. 2003 [65] and >300 µg L−1 during 1960–1997 in rivers that discharge to Barnegat Bay [66], in comparison to a similar level of 400 µg L−1 in Peconic River for Peconic Estuary [62]. In both culture and seawater microcosm studies more than 90% A. anophagefferens decline was achieved despite potentially up to 10-fold difference in Fe3^+^ concentrations in two types of media, suggesting limited variation in Fe3^+^ concentration and limited influence in shaping the outcome of bloom control in coastal water. Natural radiation (both UV and PAR, photosynthetically active radiation) likely enhance the treatment efficacy in the field. Barrington et al. examined laboratory-, mesocosm- and full-scale trials of H2O2 treatment for cyanobacterial bloom control in waste stabilization pond; they found significant synergistic effect of H2O2 addition with environmental factors (e.g. radiation) in the field trials [22]. On the other hand, H2O2 may be consumed by excessive non-target materials such as dissolved humic substances leading to decrease of treatment efficacy. Quantifying the natural fluctuation of H2O2 and OH radical concentrations and carrying out small-scale field trials with a range of H2O2 addition would likely be informative to assess the potential outcome of the full-scale field application.

In terms of environmental risk of hydrogen peroxide in natural waters, Schmidt et al. reviewed and summarized that fish and fish eggs are relatively tolerant of hydrogen peroxide, other vertebrates and mammals are much more tolerant than fish, and that microorganisms and zooplankton in aquatic ecosystem are generally less tolerant than fish or other vertebrates [29] They determined that H_2_O_2_ ≤0.7 mg L^−1^ in receiving water is not a significant threat to organisms, environmental and public health (and to be used a criterion for water quality) [29]. Indeed the reported toxicological data [28], [29], [67], [68], [69] show that 1.6 mg L^−1^ H_2_O_2_ and transient exposure (as H_2_O_2_ decays rapidly in natural waters) should exert little harm to zooplankton [28], shellfish and shellfish larvae [68], [69], fish eggs, fingerling fish and fish of cold, cool and warm water [29], [67]. Lake Koetshuis cyanobacteria bloom control with 2 mg L^−1^ H_2_O_2_ also evidenced relatively low environmental risk of this treatment to aquatic organisms, as the eukaryotic phytoplankton (including green algae, cryptophytes, chrysophytes and diatoms), zooplankton and macrofauna remained largely unaffected by the treatment [33]. Our study further evidenced relatively low risk of H_2_O_2_ treatment to marine phytoplankton in that a) phytoplankton cultures of ≥2–3 µm cell size were largely unaffected, and b) in natural seawater microcosms the diatom and the green algae were only transiently suppressed and total chlorophyll a recovered and exceeded that of the control by 72-hr of H_2_O_2_ treatment Therefore, although some eukaryotic phytoplankton could be eliminated by the H_2_O_2_ treatment at 1.6 mg L^−1^ (e.g. the target organism brown tide alga and the prasinophyte *Micromonas pusilla*), there would be very low risk for the majority eukaryotic phytoplankton, zooplankton, fish, larval fish, and other larger aquatic animals.

It is logical to think that aquatic bacterial community could be most sensitive to H_2_O_2_ treatment, as H_2_O_2_ is also used as a therapeutic agent in controlling aquaculture bacterial infection [29]. However bacteria sensitivity varies greatly and many factors including contact time, water quality and pH affect the inactivation of bacteria by H_2_O_2_ treatment [70]. Standardized bacterial culture tests showed that some bacteria had their minimum inhibitory concentration (MIC) and minimum bactericidal concentration (MBC) on the order of mg L^−1^ or higher. For example, 11 strains of 4 species of oral *Streptococci* had MIC and MBC of 3.2–14 mg L^−1^ and 3.5–28.2 mg L^−1^, respectively (for an initial 10^6^–10^7^ colony forming units per mL of logarithmic growth phase, trypticase soy broth, and 24-hr anaerobic incubation) [71]. MIC of 469–2500 mg L^−1^ were observed for cultures of *Bacillus subtilis*, *Bacillus stearothermophilus*, *Escherichia coli*, *Staphylococcus aureus Enterobacter cloacae*, *Serratia marcescens* and *Acinetobactev calcoaceticus*(for an initial ≥10^6^ CFU mL^−1^, in tryptic soy broth, 24-hr incubation) (*E. coli* had MIC of 2505 mg L^−1^) [72]. Baldry compared the bactericidal, fungicidal and sporicidal properties of hydrogen peroxide and peracetic acid and found that H_2_O_2_ to be a poor bactericide but bacteriostatic effect was achieved at and above 0.15 mM (5.1 mg L^−1^) [73]. Studies on marine bacteria were fewer; MIC was reported at 10–41 mg L^−1^ for four marine bacteria *Vibrio alginolyticus, V. harveyi, V. parahaemolyticus* and *V. vulnificus* in Mueler Hinton Broth and were 0.6–2.4 mg L^−1^ in 1.5% NaCl solution [74]. Laboratory incubation of seawater (with 10 time lower bacterial abundance as compared to the *in situ* abundance) showed that natural background levels of H_2_O_2_ (0.1–1.35 µM, 3.4–46 µg L^−1^) could cause bacterial production reduction to 25% of its control over 24-hr, and induce 1–3.4% lysogenic (due to virus) bacterial mortality [75]. Xenopoulos and Bird reported (for a humic lake in Quebec, Canada) that bacterial productivity were consistently suppressed by 3.4 mg L^−1^ H_2_O_2_ and were variably suppressed (dependent on the date and time of observation) at the exposure of as low as 0.0034 mg L^−1^ [76]. Therefore, some marine bacteria species could be very sensitive, but others such as *E. coli* very resistant. Nevertheless, bacteria are expected to inoculate from surrounding waters and quickly reproduce and repopulate the treated water once H_2_O_2_ is sufficiently dissipated after treatment. Thus, long lasting impacts are not expected.

Cyanobacteria community could be most severely affected by H_2_O_2_ treatment, as was seen in the field work of Matthij et al. where 2 mg L^−1^ H_2_O_2_ eradicated cyanobacterial bloom of *Planktothrix agardhii* in the freshwater lake [33]. However, cyanobacterial sensitivity to H_2_O_2_ also varied substantially among the species. For example, EC_50_ for photosynthetic yield (3-hr H_2_O_2_ exposure) were 0.3–0.4 mg L^−1^ for *Microcystis aeruginosa* and *Trichormus variabilis* but were 4-fold higher at 1.1–1.7 mg L^−1^ for *Synechococcus nidulans* and two other cyanobacteria species [42]. Schrader et al. reported a lowest complete inhibitory concentration (LCIC) of 100 µM for sodium carbonate peroxide (equivalent to 32.5 µM or 1.1 mg L^−1^ H_2_O_2_) for off-flavor producing cyanobacteria *Oscillatoria cf. chalybea* but it was 10-fold higher (equivalent to 325 µM or 11 mg L^−1^ H_2_O_2_) for *Anabaena sp* LP691 [23]. Kay et al. reported more than 85% bleach of chlorophyll in cyanobacteria *Raphidiopsi*s sp. after 48-hr exposure to 200 µM H_2_O_2_ at 90 µmol m^−2^ s^−1^ but no significant bleach was observed in *Anabaena sp* under the same condition [77]. Observing substantial species variability in H_2_O_2_ tolerance among cyanobacteria, Schrader et al. concluded that H_2_O_2_-based algaecide “may be less disruptive to the aquatic ecosystem by not eliminating all of the cyanophytes so quickly as to create low dissolved oxygen levels which could stress and kill fish” [23]. In our seawater microcosm study the cyanobacterial population was not completely eliminated, but reduced about 50% by 72-hr of H_2_O_2_ addition (as compared to the control without H_2_O_2_ addition). Furthermore, the most sensitive cyanobacteria species, though eliminated by H_2_O_2_ treatment, likely get re-inoculated from surrounding water via natural water exchange, while the more resistant ones would persist and repopulate the treated water.

Hydrogen peroxide is rapidly decomposed into water and oxygen [17], [18], [19], [20], and does not produce persistent chemical residues. Cooper et al [78]. observed the half-life of hydrogen peroxide in lake water at 4.4, 4.7, 6.4, 19.1 and 58.7 hr in unfiltered, 64 µm filtered (zooplankton removed), 12 µm filtered (large algae removed), 1 µm filtered (small algae removed), and 0.2 µm filtered (bacteria removed) waters, respectively. Matthij et al. found that hydrogen peroxide applied to Lake Koetshuis at 2 mg L^−1^ was degraded to 0.7 mg L^−1^ after one day and to below detection limit (∼ 0.1 mg L^−1^) after two days [33]. Thus, we expect the concentration of H_2_O_2_, if applied at 1.6 mg L^−1^ to natural seawater, to be reduced to the natural background level within a couple of days due to decomposition and simultaneous dilution. The proposed concentration is indeed quite low as compared to a) the proposed receiving water quality criterion of 0.7 mg L^−1^ [29], b) the natural occurrence in rain water at e.g. 5–25 μM H_2_O_2_ in North Carolina [79] or up to 57 µM (∼ 2 mg L^−1^) at Bermuda Atlantic Time Series Stations [17], and c) up to hundreds nM background existence in surface seawater [15], [16]. The decomposition of H_2_O_2_ potentially improves water quality by oxygenating the water column, facilitating dissolved organic matter degradation [80], and even inactivating algal bloom toxins [21]. Further, in natural waters, UV-light could have synergistic effect with H_2_O_2_ [18], thus even lower dose than 1.6 mg L^−1^ H_2_O_2_ may be effective in brown tide bloom control.

### Conclusions

Harmful algal bloom has become an increasingly important issue to the economic development and environmental sustainability, such that it demands both direct control methods and long term prevention measures. This study evidenced, from culture and microcosm studies, that 1.6 mg L^−1^ H_2_O_2_ effectively removed brown tide even at peak bloom intensity, and the other phytoplankton of >2–3 µm cell size remained largely unaffected by the treatment. Thus hydrogen peroxide is proposed as a natural and environmentally friendly agent potentially useful for brown tide control.
